# Progress in Surgery of the Stomach

**Published:** 1902-06-07

**Authors:** 


### Progress in Surgery of the Stomach.

**Gastric Ulcer.**—Maunsell [1] draws attention to the following points in forming an early diagnosis in cases of perforated gastric ulcer:—1. The pain which begins in the epigastrium spreads, but does not shift its position. 2. There is no pain on micturition, which is a frequent sign of peritonitis of the lower abdomen. 3. Thirst is not intense, and there is no flinging about, etc., as in hæmorrhage. 4. There may be no distension in muscular subjects, the tympanites and free gas being shown only by encroachment on the thoracic area. 5. The pulse cannot be relied upon in forming an early diagnosis. 6. The statement sometimes made that a "stomach note" excludes perforation is unwarrantable, as a perforated and collapsed stomach are by no means synonymous terms. 7. Liver dulness is diminished or absent in almost every case. Mr. Maunsell advocates douching combined with careful sponging, and closure of the abdomen without drainage. M'Ardle [2] says there are two positions in the stomach in which he would not suture at all—(1) where the œsophagus joined it, and (2) where a fixed pylorus prevented the procedure being properly carried out. In these cases the abdominal cavity should be tamponed. Parsons [3] considers the steady increase in the pulse rate is a most valuable sign, and one not masked by morphia. The temperature is no guide. Power [4] points out the difficulty of making an exact diagnosis in many acute abdominal lesions. There is, however, no doubt that an abdominal exploration is required immediately in cases of acute pain with shock, tenderness and rigidity in the upper abdomen, vomiting, with the history of even the mildest dyspeptic symptoms, for these signs should incline the opinion toward perforation rather than towards any other acute condition high up in the abdominal cavity. Without pain acute epigastric symptoms cannot be caused by a perforated gastric ulcer, and pain is therefore the most important symptom of perforated gastric ulcer; but there must be in addition evidence of peritoneal shock—*i.e.*, elevation of pulse, normal or subnormal temperature, rigidity of muscles, and local tenderness. Whyte [5] says the chief indications for operation in gastric ulcer are:—(1) Stricture of pylorus; (2) ulcers, especially in the neighbourhood of the pylorus, which defy internal treatment, and cause intolerable suffering; (3) repeated small hæmorrhages; (4) severe hæmorrhages threatening life, which may make operation necessary as the only hope; (5) perigastric adhesions after ulcers, when they cause great discomfort; (6) perforations, for which operation is urgently indicated within the first 12 hours. Spence [6] says perforated gastric ulcer is more common in Europe than in America, because of the existence in Europe of a peasant class entirely unknown in America. Of 281 cases 199 were females, and of 237 cases 125 were 25 years of age or younger. Perforations are more common on the anterior surface and near the cardia, whereas ulcers are most common on the posterior surface and near the pylorus. The lesser curvature is a more frequent site of both ulcer and perforation than the greater curvature. Horsford [7] records a case of perforated gastric ulcer simulating appendicitis, and other cases of perforated ulcer are recorded by Brown,[8] Wingfield and Langdon,[9], Wheeler,[10] Lacy and Whipple,[11] Pye-Smith,[12] Stewart,[13] and others.

**Gastric Hæmorrhage.**—Mayo Robson [14] says it is impossible to diagnose the size of the vessel perforated either from the amount of blood lost or the length of survival. A probable diagnosis may, however, often be arrived at in hematemesis from ulcer, by a study of the previous history, especially with regard to the site of pain, the time of onset after food, and the influence of posture on it, as well as by the way the pain radiates. If medical treatment and rest properly carried out are not successful in arresting the bleeding in a few hours, or if after being arrested it recurs, we should be driven to the conclusion that a large vessel is perforated, and the question of operation for the immediate arrest of the bleeding by direct treatment should be considered. At present, with the exception of Dieulafoy, who advocates operation during the first bleeding if as much as half a litre of blood is lost, all other surgeons who have written on the subject agree that general means ought to be relied on during and after a first attack, as in from 93 to 95 per cent. of cases such treatment succeeds, and until our means of diagnosis as to size of vessel injured is rendered more reliable we must assent to this rule, but after a second bleeding Robson would advise operation during the course of the hæmorrhage if the patient can stand it, or as soon after as his condition will permit the operation to be done, for experience tells us that further hæmorrhages are almost certain to occur unless preventive means are adopted. After the abdomen in opened, the stomach may be emptied by pressing the contents into the duodenum. The surface of the stomach should then be carefully examined for evidence of ulceration, but if no indication of this sort be obtainable, the stomach must be drawn well forward and opened either by a vertical incision, which gives less hæmorrhage, or by an incision in the long axis of the stomach, which gives a much better view of the interior, which should then be systematically examined while the stomach is held open with forceps. If the posterior wall of the stomach cannot be efficiently examined, a slit may be made in the omentum and two or more fingers pushed through it and employed to invaginate the posterior wall. If no ulcer is then found the duodenum should be explored, not only by the finger, but by invagination. If no ulcer be found anywhere and the bleeding proves to be capillary or from small undiscoverable ulcers, gastro-enterostomy should be performed; but if an ulcer be discovered, and it be possible to excise it, that operation should be done. After excision of the ulcer and ligature of the bleeding vessels the edges of the wound are brought together by sutures. Should the ulcer be discovered in some region where excision is impracticable—for instance, near the cardiac end—the actual cautery may be employed. In cases of this kind, gastro-enterostomy is also advisable by way of setting the stomach at rest.

**Cancer of the Stomach.**—Moynihan[15] contends that the surgery of cancer of the stomach should be brought into line with the surgery of malignant disease elsewhere by the removal of the lymphatic systems associated with the growth. That this is necessary is shown by the statistics of Hemmeter that recurrences occur in 99 per cent. of cases submitted to operation. The lymphatic glands of the stomach are situated chiefly along the vessels: (1) The coronary group lie along the lesser curvature. These glands are continuous at the origin of the coronary artery with those along the upper border of the pancreas. (2) The hepatic group lie along the hepatic artery and some of its members along the pyloric artery. The glands of the greater curvature lie along the right gastro-epiploic artery. At the pylorus they are numerous and close together, and pass behind the stomach to the head of the pancreas. The lymphatic vessels draining in these glands pass very obliquely into the walls of the stomach. Three chief lymphatic areas of the stomach may be described. An area along the lesser curvature, an area along the greater curvature, and an area comprising the greater tuberosity of the stomach extending up to the œsophagus and on to the greater curvature as far as the limit of supply of the left gastro-epiploic artery (as far as the gastro-splenic omentum). In cases of malignant disease of the pyloric portion of the stomach, the growth extends in the direction of the curvatures, and the glands of the first two groups are affected, but the glands of the third group and the area which they drain escapes. This latter area may be looked upon as an area apart, as one into which extension rarely occurs, and as one, therefore, the lymphatic vessels and glands of which remain healthy. The term "isolated area" might well be employed to describe it. Moyinham points out that malignant disease most often occurs near the pylorus, and in order to ensure complete removal of the primary growth of the infiltrated lymphatic vessels, and of the glands into which those vessels drained, it is necessary to remove the stomach as far up on the lesser curvature as the point of abatement of the coronary artery, and on the greater curvature as far as the gastro-splenic omentum, and to remove the first portion of the duodenum. Meyer[16] advocates the elastic ligature (McGraw's method) in the performance of gastro-enterostomy.

[1] Dublin Jour. Med. Sci., July 1901. p. 60. [2] Ibid. [3] Ibid. [4] Med. Chron., June, 1901, p. 194. [5] Scottish Med. and Surg. Jour., Aug., 1901, p. 167. [6] Brooklyn Med. Jour., Jan., 1902, p. 7. [7] Lancet, Feb. 15, 1902, p 441. [8] Lancet, Sept. 14. 1901, p. 731. [9] Lancet, Aug. 17, 1901. p. 451. [10] Lancet, Oct. 26. 1901, p. 1121. [11] Lancet, Feb. 15, 1902, p. 444. [12] Quarterly Med. Jour., Nov., 1901, p. 1. [13] Brit. Med. Jour., March 1, 1902, p. 510. [14] Scottish Med. Jour., March, 1901, p. 205. [15] Lancet, Nov. 16, 1901, p. 1339. [16] Med. Rec., Jan. 25, 1902, p. 133.

